# *Wohlfahrtiimonas chitiniclastica* Infections in 2 Elderly Patients, Hawaii, USA

**DOI:** 10.3201/eid2203.151701

**Published:** 2016-03

**Authors:** Masayuki Nogi, Matthew J. Bankowski, Francis D. Pien

**Affiliations:** University of Hawaii at Manoa John A. Burns School of Medicine, Honolulu, Hawaii, USA (M. Nogi, M.J. Bankowski, F.D. Pien);; University of Hawaii at Manoa, Honolulu (M. Nogi, M.J. Bankowski, F.D. Pien);; Diagnostic Laboratory Services, Inc., Aiea, Hawaii, USA (M.J. Bankowski)

**Keywords:** Wohlfahrtiimonas chitiniclastica, sepsis, skin, soft tissue, infection, Hawaii, bacterial infection, bacteria

**To the Editor:** We describe 2 cases of *Wohlfahrtiimonas chitiniclastica* sepsis and skin and soft tissue infections in 2 elderly patients; 1 case was fatal. Both patients lived in poor hygienic conditions in Hawaii, USA. 

The first case occurred in a 72-year-old man with history of stroke and deafness. After being unattended for 3 days, he was found unconscious on the floor of his home. He was hypotensive, bradycardic, and hypothermic. Maggots were crawling out of an umbilical wound and were present in a 2 × 3 cm laceration on his right dorsal foot. His leukocyte count was 2.4 × 10^3^ cells/μL with 42% band cells, creatinine level was 2.5 mg/dL, and lactic acid level was 1.8 mg/dL. Aerobic and anaerobic cultures of blood collected at hospital admission grew *Escherichia coli* and *W. chitiniclastica* within 12 hours. Initial treatment consisted of intravenous piperacillin/tazobactam (4.5 g every 6 h), intravenous clindamycin (900 mg every 8 h), and intravenous vancomycin (1,000 mg every 12 h). The patient died from septic shock on his second hospital day. *W. chitiniclastica* was identified by using 16S rRNA sequencing (MicroSeq 500 16S rDNA Bacterial Identification Kit; Applied Biosystems, Foster City, CA, USA) and analyzed by using RipSeq mixed DNA interpretation software (iSentio Ltd., Bergen, Norway). A 100% match with *W. chitiniclastica* type strain H100 (GenBank accession no. HQ407275) was observed. Antimicrobial drug–susceptibility testing was performed by using a microdilution method (MicroScan Dried Overnight Gram-Negative Panel; Siemens Medical Solutions, Malvern, PA, USA). The isolate was sensitive to all drugs tested, including classes of penicillin, cephalosporin, fluoroquinolone, carbapenem, tetracycline, and aminoglycoside.

The second case occurred in a 69-year-old homeless woman with a history of right hemiparesis from a ruptured cerebral aneurysm. She reported having had sacral pain and painful urination during the week before admission. Physical examination revealed stable vital signs, disheveled appearance, and multiple purulent decubitus ulcers in her sacral area. Her leukocyte count was high (20.9 × 10^3^ cells/μL). Urinalysis revealed pyuria, positive nitrates, and moderate leukocyte esterase, indicative of a urinary tract infection. Two blood cultures and urine culture were obtained at admission. She was given intravenous ceftaroline fosamil (600 mg every 12 h) to treat the urinary tract and decubitus ulcer infections. She then underwent surgical debridement of her decubitus ulcers, where tissue from a deep wound was obtained for aerobic and anaerobic culture. The deep wound culture grew polymicrobial flora that included *W. chitiniclastica*, *Staphylococcus aureus*, *Aeromonas* spp., *S. simulans,* and *Bacteroides fragilis*. The anaerobic bottle from both blood cultures grew a gram-negative anaerobic bacillus, *Anaerobiospirilum succinicproducens*. In addition, *Proteus mirabilis* was isolated from a urine culture. These culture results prompted a change in the patient’s antimicrobial drug regimen to intravenous meropenem (1 g every 8 h), which the patient received for 12 days. She responded well and was discharged and prescribed oral amoxicillin/clavulanate (875 mg/125 mg every 12 h) for 3 weeks, for what would amount to a 34-day course of antimicrobial treatment since her hospital admission. *W. chitiniclastica* was identified by using 16S rRNA sequencing (MicroSeq 500 16S rDNA Bacterial Identification Kit). A 100% match with *W. chitiniclastica* type strain H100 (GenBank accession no. HQ407275) was observed. Antimicrobial drug–susceptibility testing results were the same as those observed for the previously described patient.

*W. chitiniclastica* is a short, gram-negative, facultative anaerobic, and motile gammaproteobacterium with strong chitinase activity. It was isolated from the homogenated third-stage larvae of the *W. magnifica* fly (*1*) (Figure). This fly has been reported as the cause of myiasis in live vertebrates in Spain, France, Hungary, Turkey, Egypt, Iran, and Korea (*2*); its distribution is known to be progressively expanding, in part because of its broad adaptation capacities. Reported cases of human bacteremia have been mainly from Europe and South America; patients included a 60-year-old homeless woman in southeastern France (*3*), a 70-year-old homeless man with alcoholism in Argentina (*4*), and an 82-year-old woman in the United Kingdom (*5*). A skin and soft tissue infection was reported in a child with orofacial gangrene (noma) in Niger (*6*), and an osteomyelitis case was reported in India (*7*). The northernmost region from which a case has been reported is Estonia, where a 64-year-old man with chronic foot gangrene was coinfected with *W. chitiniclastica* and *Myroides odoratimimus* (*8*). Cases from the United States include septicemia in a deer in Michigan (*9*) and a leg wound infection in a 26-year-old man in New York (*10*).

**Figure:**
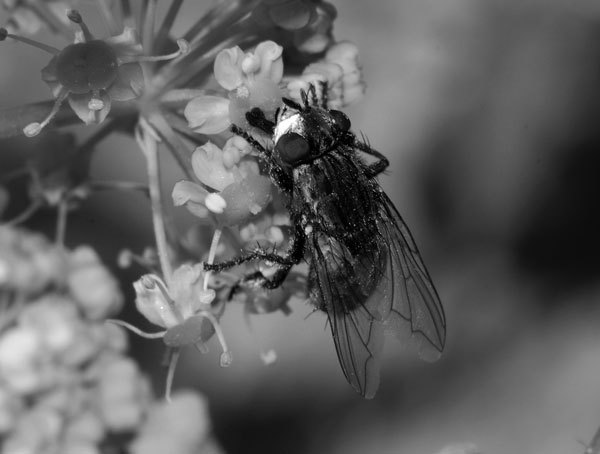
Adult *Wohlfahrtiimonas magnifica* fly. Image courtesy of Joaquim Alves Gaspar, Wikimedia Commons.

For the 2 patients in Hawaii, maggots were not collected, and we could not identify the specific fly species. In the first patient, *W. chitiniclastica* was clinically relevant because it was isolated from blood culture and maggots were observed in his wound. However, the coexisting *E. coli* infection may have played a critical role in the patient’s death. The second case was nonfatal, and we cannot determine the clinical relevance of *W. chitiniclastica* because it was isolated from a polymicrobial wound in which no maggots were observed and because *A. succinicproducens* was isolated from blood culture*.* Even so, these reports should help increase awareness of this specific type of infection related to myiasis in homeless and hygiene-deficient patients in the United States.
